# Respiratory Support Strategies for Surgical Neonates: A Review

**DOI:** 10.3390/children12030273

**Published:** 2025-02-24

**Authors:** Piero Alberti, Niyi Ade-Ajayi, Anne Greenough

**Affiliations:** 1 Department of Women and Children’s Health, School of Life Course Sciences, Faculty of Life Sciences and Medicine, King’s College London, London SE5 9RS, UK; piero.alberti1@nhs.net (P.A.); niyi.ade-ajayi@nhs.net (N.A.-A.); 2 Department of Paediatric Surgery, King’s College Hospital, Denmark Hill, London SE5 9RS, UK

**Keywords:** paediatric surgery, neonatal ventilation, oesophageal atresia, tracheoesophageal fistula, congenital diaphragmatic hernia, congenital lung malformations, gastroschisis, exomphalos

## Abstract

Neonates with congenital conditions which require surgical management frequently experience respiratory distress. This review discusses the management of pulmonary complications and the respiratory support strategies for four conditions: oesophageal atresia-tracheoesophageal fistula (OA-TOF), congenital diaphragmatic hernia (CDH), congenital lung malformations (CLM), and anterior abdominal wall defects (AWD). Mechanical ventilation techniques which can reduce the risk of ventilator-induced lung injury (VILI) are discussed, as well as the use of non-invasive respiratory support modes. While advances in perioperative respiratory support have improved outcomes in infants with OA-TOF, managing respiratory distress in premature OA-TOF neonates remains a challenge. In CDH infants, a randomised trial has suggested that conventional ventilation may improve outcomes compared to high-frequency ventilation. Echocardiographic assessment is essential in the management of CDH infants with pulmonary hypertension. Lung-protective ventilation settings may lower the rate of postoperative complications in symptomatic CLM infants, but there remains debate regarding the choice of expectant versus surgical management in neonates with asymptomatic CLMs. Infants with AWDs can require ventilation due to pulmonary hypoplasia, but the effects of this on their long-term respiratory health are poorly understood. As surgical techniques continue to evolve and novel ventilation techniques become available, prospective multi-centre studies will be required to define the optimal respiratory support strategies for neonatal surgical conditions that affect lung function.

## 1. Introduction

Infants with congenital surgical conditions affecting the respiratory system can require support in the pre- and intraoperative periods and in postoperative recovery [1,2]. While lifesaving, the use of invasive mechanical ventilation (MV) in the neonatal period is associated with the risk of ventilator-induced lung injury (VILI), including bronchopulmonary dysplasia (BPD) and other forms of chronic respiratory morbidity [3,4].

Studies have investigated the benefits of various modes of respiratory support in surgical neonates, including non-invasive respiratory support as well as alternatives to conventional invasive MV [2,3]. This review focuses on advances in respiratory support for four surgical conditions affecting neonatal lung function: oesophageal atresia and tracheoesophageal fistula (OA-TOF), congenital diaphragmatic hernia (CDH), congenital lung malformations (CLM), and anterior abdominal wall defects (AWD).

### Principles of Respiratory Support in the Surgical Neonate

All neonates are affected by a degree of alveolar immaturity due to the ongoing development and maturation of alveoli into childhood and adolescence [5]. Alveolar immaturity is most pronounced in babies born extremely premature (<28 weeks of gestation) or at extremely low birth weight (ELBW) [3,4]. Prematurity is also associated with reduced lung compliance due to insufficient production of surfactant, as in respiratory distress syndrome (RDS). In surgical neonates, respiratory function may be further compromised by associated lung hypoplasia, aspiration of oral or gastric contents (OA-TOF), and compression of hypoplastic lungs by lung masses (CLM) or intrathoracic abdominal contents (CDH) [1].

Neonatal respiratory support strategies are used to maintain the lung at a volume that ensures adequate oxygenation and removal of CO_2_ while minimising the driving mechanisms of VILI. The latter include alveolar overdistension (volutrauma), shear stress due to collapse and re-recruitment of alveoli (atelectrauma) and overexposure to oxygen (biotrauma) [1]. Studies have explored the feasibility of managing pre- and postoperative surgical neonates with non-invasive support strategies such as humidified, high-flow nasal cannulae (HHFNC), continuous positive airway pressure (CPAP) or nasal intermittent positive pressure ventilation (NIPPV) [3]. While conventional invasive ventilation remains the mainstay of treatment for most neonates requiring intubation, studies have investigated the potential benefits of open-lung strategies that reduce atelectrauma by distending the lungs at uniform mean airway pressure (MAP) such as high-frequency oscillatory or jet ventilation (HFOV/HFJV). Respiratory support for surgical neonates may also be optimised by the use of synchronised ventilation modes.

## 2. Oesophageal Atresia-Tracheoesophageal Fistula

Oesophageal atresia (OA), with or without tracheoesophageal fistula (TOF), occurs in between 1 in 2000 to 1 in 4000 live births [6]. Whilst around a third of cases are identified from findings on antenatal ultrasonography (polyhydramnios, distended oesophageal pouch, absent stomach bubble), most are diagnosed after birth from a clinical picture of hypersalivation, choking on feeds, and failure to pass an appropriately sized nasogastric (NG) tube into the oesophagus [6,7]. The diagnosis is then confirmed on a chest radiograph demonstrating the coiled NG tube within the proximal oesophageal pouch.

OA-TOF was a universally fatal condition until the 1930s [8]. Survival from OA-TOF then improved from an approximately 50% rate in high-resource specialist centres in the 1950s to a current survival above 85% [8,9]. In term neonates without major cardiac anomalies and with access to specialist neonatology and surgical care, survival from OA-TOF has been above 90% since the 1980s [10,11]. Alongside advances in surgical technique, increased capacity for and improvement in neonatal respiratory support strategies have been the key drivers of this reduction in mortality [12].

As most OA-TOF neonates are born at term and without associated lung disease, patients can usually be allowed to breathe spontaneously and only require invasive MV during surgery and in the postoperative period. Respiratory problems, however, arise in patients with co-existing congenital heart disease, which is observed in over 30% of patients as part of the VACTERL association, defined as presence of at least three out of the five additional features: vertebral anomalies, anorectal malformations, congenital cardiac defects, renal anomalies, limb defects [13]. Respiratory support is also challenging in OA-TOF neonates born prematurely or at birth weight below 2 kg, and those with large (diameter > 3 mm) and/or pericarinal fistulae [13,14].

### 2.1. Preoperative Optimisation

The main aspect of preoperative management for OA-TOF neonates is prevention of aspiration. This is achieved by exclusion of oral feeds, prone placement with head elevation, and drainage of the upper oesophageal pouch by intermittent suction or continuous low-pressure suction through a Replogle tube. In low-resource settings, early cervical oesophagostomy may be performed to reduce the risk of aspiration while awaiting transfer for definitive repair [15]. This procedure, however, can be a significant surgical and anaesthetic challenge even in a specialist setting.

**Figure 1:**
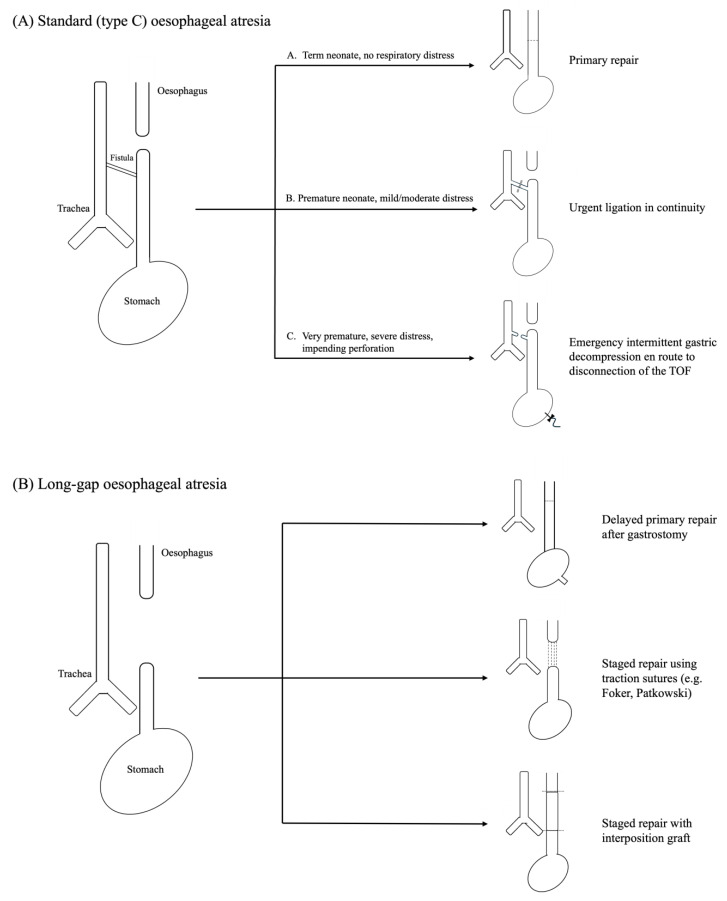
Surgical strategies in OA-TOF.

In term neonates who are breathing spontaneously and are not fed, OA-TOF is not initially an acute surgical emergency. Over hours to days after birth, however, repeated aspiration of gastric acid into the tracheobronchial tree may result in chemical pneumonitis and subsequent pulmonary hypertension. The trend in the management of term-born OA-TOF neonates currently favours delayed surgery to allow for thorough preoperative assessment and selection of the most appropriate anaesthetic and surgical teams. By contrast, preterm OA-TOF neonates can require emergency invasive respiratory support due to reduced lung compliance. Intubation and ventilation of OA-TOF infants is frequently complicated by significant air leaks and, if the airway is in communication with the distal oesophageal pouch, by the risk of gastric distension from the use of MV and positive end-expiratory pressure (PEEP) [7]. The complexity in these patients lies in balancing the risk of inadequate respiratory support with that of gastric perforation, which is higher in those patients who also have duodenal atresia and an associated gastric outlet obstruction. Any increase in the respiratory support requirements of these patients is worrisome and requires coordinated proactive interventions by the neonatology, anaesthetic, and paediatric surgical teams. Placement of a decompressing gastrostomy prior to repair of the tracheoesophageal fistula effectively creates an iatrogenic bronchocutaneous fistula with an associated low-pressure air leak, which may compromise ventilation [16,17]. In this scenario, the strategy favoured in our unit is stabilisation of the patient by emergency endotracheal intubation and transabdominal insertion of a butterfly needle into the stomach. After this is secured by tape, intermittent decompression is undertaken as required while preparing for closure of the tracheoesophageal fistula in the operating theatre or at the cotside in the NICU. Ligation without disconnection is the preferred surgical option for emergency fistula closure in more mature infants, as this preserves oesophageal length and is important, in due course, for a tension-free oesophageal anastomosis. However, as ligation in continuity only provides a finite window of time before re-fistulation occurs, sacrificing oesophageal length by disconnecting the fistula at the time of the first intervention is recommended in very premature OA-TOF neonates. Repair of the atretic oesophagus at a later stage is prudent in these patients as they require prolonged invasive respiratory support before being well enough for oesophageal reconstruction (Figure 1A).

Less common and/or more complicated variants are not discussed. When technically feasible without undue tension, primary repair is the preferred approach to management of long-gap OA. However, traction-based staged repair or oesophageal replacement by gastric transposition or interposition graft may be required in cases with particularly wide gaps between the oesophageal pouches.

### 2.2. Intraoperative Considerations

In a sub-analysis of the multicentre NECTARINE audit of paediatric anaesthesia in Europe, which included over 100 patients, OA-TOF surgery was found to be associated with a 40–50% rate of intraoperative events requiring ventilatory intervention [18]. The vast majority of these were accounted for by transient desaturations due to displacement of the endotracheal tube or minor lung contusion due to intraoperative lung retraction [7].

Intraoperative respiratory management of OA-TOF depends on patient- and procedure-specific factors. While most cases are amenable to primary repair (open, thoracoscopic and, recently, robotic), the 10% of cases of long-gap oesophageal atresia require more complex strategies and procedures [19]. These include delayed primary repair and staged repairs involving traction of oesophageal pouches (e.g., Foker external traction, Kimura advancement, Patkowski thoracoscopic internal traction) or oesophageal replacement via gastric pull-up, jejunal interposition, or colonic interposition (Figure 1A) [20,21,22].

A combination of rigid or flexible laryngo-tracheobronchoscopy (TBS) and oesophagoscopy can be used to confirm the diagnosis and guide respiratory support during surgery [23]. TBS can aid characterisation of associated upper airway anomalies (e.g., laryngotracheal clefts, subglottic stenosis, tracheomalacia) and visualisation of the TOF to guide endotracheal tube placement [23,24]. In a recent international survey, however, only 50% of respondents used TBS as part of routine preoperative assessment of OA-TOF patients [25,26,27]. Moreover, the performance of rigid TBS in neonates is increasingly becoming a skill specific to paediatric respiratory physicians and otolaryngologists rather than general paediatric surgeons, especially in tertiary centres of high-income countries [23,27].

Thoracoscopic repair of OA-TOF may be associated with an increased risk of intraoperative hypercapnia and/or respiratory acidosis due to systemic absorption of the insufflating CO_2_ [28,29,30,31,32,33]. Whilst a pilot randomised controlled trial (RCT) did not identify significant differences in intraarterial p_a_O_2_, p_a_CO_2_, or pH in open versus thoracoscopic repair, only ten patients were included in the study [31]. In a retrospective study, thoracoscopic (*n* = 14) and open (*n* = 62) repair were both associated with hypercapnia and acidosis, although neonates undergoing thoracoscopic repair developed lower arterial pH than those having open surgery [33]. No study, however, has reported a significant correlation between intraoperative acidosis and rates of surgical complications or the duration of postoperative ventilation. While a study using near-infrared spectroscopy (NIRS) suggested that CO_2_ absorption might lead to a reduction in cerebral O_2_ saturation (cSO_2_) in OA-TOF neonates, a later study found that cSO_2_ values remained within safe limits if thoracoscopy was performed at lower (5 mmHg) CO_2_ pressures [29,32]. No studies have reported long-term neurodevelopmental outcomes after open versus thoracoscopic repair of OA-TOF [30]. Alternative modes of MV have been explored as potential strategies to reduce arterial gas derangement in minimally invasive OA-TOF surgery [34]. Single-centre retrospective studies showed that thoracoscopic OA-TOF repair in HFOV-ventilated neonates was associated with improved CO_2_ elimination without an increased complication rate [28,35].

### 2.3. Postoperative Support

Elective ventilation after repair of OA-TOF can reduce the tension on the anastomosis by stabilising the neck and eliminating active swallowing [36]. A meta-analysis of retrospective studies (*n* = 165) showed that the combination of muscle paralysis, positive pressure MV and neck flexion was associated with reduced rates of anastomotic leak after OA-TOF surgery [37]. The trend over the last two decades has been to favour early extubation, aiming for a quicker progression from trans-anastomotic tube (TAT) to oral feeds and prevention of complications such as ventilator-associated pneumonia [38,39,40]. In the NECTARINE audit, the reported median duration of postoperative MV was five days [18]. The factors affecting the duration of postoperative ventilation in OA-TOF infants are the assessment of the risk of anastomotic leak, which depends on the tension and blood supply of the anastomosis and is highest in cases of long-gap OA, sedation requirements, which are reduced after thoracoscopic surgery and by the use of thoracic caudal epidural catheters, and delays in extubation due to secondary upper airway anomalies such as laryngotracheal clefts, tracheomalacia or vocal cord palsy, which may occur in more than 30% of patients [6,41,42].

The optimal timing of extubation following repair of OA-TOF is controversial. In a single-centre review, extubation within 24 h of surgery (*n* = 16) was found to be as safe as delayed extubation (*n* = 18), whereas infants extubated in the operating theatre (*n* = 12) were found to be at significantly higher risk of needing reintubation due to respiratory distress [43]. Emergency reintubation of post-operative OA-TOF neonates represents a challenging procedure, especially in patients with a tight anastomosis, and is associated with an increased risk of complications [6]. Studies have therefore investigated whether non-invasive respiratory support modes may facilitate safe early extubation in OA-TOF infants. One report suggested that post-extubation bridging with CPAP may be safe, but only included ten infants, whilst another (*n* = 25) found that NIPPV and HHFNC were associated with increased risk of anastomotic leak and mediastinitis [44,45,46]. In a recent study (*n* = 32), post-extubation CPAP, NIPPV, or HHFNC after 6–7 days of invasive MV were not associated with a higher leak rate compared to spontaneous breathing [47]. Given the small sample sizes and retrospective nature of existing evidence, larger prospective trials are needed to delineate the mode of postoperative respiratory support that may minimise both respiratory and surgical complications following OA-TOF repair.

## 3. Congenital Diaphragmatic Hernia

Congenital diaphragmatic hernia (CDH) occurs in around 1 in 4000 live births and is the neonatal surgical condition in which respiratory support is most complex. Lung hypoplasia and pulmonary hypertension (PH) are the key factors driving the 30% mortality observed in CDH patients [48,49]. In an analysis of over 1400 patients in the CDH Study Group (CDH-SG) international registry, PH was observed in 86.5% of cases and was an independent predictor of mortality at thirty days of life [50,51]. In addition, left ventricular (LV) hypoplasia due to compression of the mediastinum by abdominal contents, leading to LV systolic and diastolic dysfunction, can further compromise respiratory function in CDH neonates [52].

### 3.1. Antenatal Management

Most cases of CDH in high-resource settings are now identified on antenatal ultrasound [53]. Several fetal parameters have been investigated as potential prognostic indices that may guide parental counselling, specialist centre referral and fetal intervention. Herniation of the liver into the thorax, reduced lung area to head circumference ratio (LHR) and reduced absolute or relative lung volumes on fetal MRI have all been shown to correlate significantly with the requirement for postnatal extracorporeal membrane oxygenation (ECMO) and mortality [54,55,56,57,58]. Whilst a study of fetal tracheal occlusion after hysterotomy did not show survival benefits [59], the TOTAL RCT demonstrated that fetal endoscopic tracheal occlusion (FETO) by fetoscopic tracheal balloon placement at 27 to 29 weeks of gestation significantly reduced mortality in infants with severe left-sided CDH (liver herniation and observed/expected LHR ≤ 25%), leading to reduced requirement for ECMO, improved survival to discharge and improved survival to six months of age compared to expectant management [60]. No survival benefit was instead observed among infants with moderate CDH who had FETO at 30 to 32 weeks of gestation [61]. In both patient groups, FETO was associated with a significantly increased incidence of preterm, prelabour rupture of membrane and preterm birth [60,61]. As a result, FETO is currently only recommended for fetuses with severe left-sided CDH.

Since FETO is selectively performed in severe CDH cases, subsequent studies observed that CDH neonates who had been treated by FETO were significantly more likely to need a prosthetic patch for repair of their diaphragmatic defect and had significantly longer durations of MV, supplemental oxygen and NICU stay [62]. Some studies suggested that FETO-treated neonates may be at risk of clinically significant tracheomegaly or tracheomalacia [63]. Whilst single-centre retrospective reviews of radiographic measurements observed larger tracheae in FETO-treated neonates, this was not associated with increased respiratory support requirements or mortality. In addition, gradual remodelling of the trachea from a cystic to a fusiform shape was demonstrated in later postnatal life [64,65]. In a multi-centre cohort study, tracheal diameter in FETO-treated CDH infants (*n* = 287) was on average 31.3% wider than in CDH infants not treated with FETO. Most cases of tracheomalacia also resolved spontaneously with less than 1% of families reporting persistent debilitating respiratory issues [66].

### 3.2. Perinatal Stabilisation

In antenatally diagnosed CDH neonates, both the European CDH Consortium (CDH EURO) and the Canadian CDH Collaborative recommend intubation at birth to minimise insufflation of herniated bowel, with monitoring of pre- and post-ductal SO_2_, lung compliance and tidal volumes. In CDH patients presenting with intrathoracic distension of the stomach, emergency gastric decompression by insertion of a NG tube or percutaneously may be required [67]. A single-centre case series reported that some neonates with mild CDH (abdominal liver, O/E LHR > 50%) could be allowed to breathe spontaneously without adverse effects [68].

There is no consensus on the optimal fraction of supplemental oxygen (FiO_2_) for stabilisation of CDH neonates. Low P_a_O_2_ is associated with worsening of PH, while excessive F_i_O_2_ may blunt the response to pulmonary vasodilators and lead to cerebral hyperoxia [48]. A retrospective cohort study (*n* = 68) found that stabilising patients on a lower F_i_O_2_ (0.5) did not affect mortality or ECMO requirements compared to a starting F_i_O_2_ of 1.0 [69]. The current recommendation from the CDH EURO Consortium is to start resuscitation at an F_i_O_2_ of 0.5 and if necessary titrate the F_i_O_2_ upwards to maintain target pre- and post-ductal SO_2_ ranges of 80–95% and >70%, respectively [48,69].

The use of neuromuscular blockers for intubation in the delivery suite is not recommended as a prospective study (*n* = 15) found it to be associated with decreased lung compliance [70]. Immediate clamping of the umbilical cord may also worsen PH by reducing cardiac preload while the physiological postnatal increase in pulmonary venous return is delayed, leading to hypoxia and pulmonary vasoconstriction [71]. Delaying cord clamping until the lungs have fully aerated, known as physiological-based cord clamping (PBCC), has been found to improve pulmonary blood flow in animal models and has been shown to be feasible and safe in CDH patients [72,73]. The impact of PBCC on mortality, PH rates, ECMO requirement, and duration of MV in CDH is currently being investigated in the multi-centre PinC trial [74].

Various prognostic algorithms for CDH neonates have been designed by combining perinatal arterial blood gas measures, such as the Wilford Hall/Santa Rosa index or the CDH study group index. The best oxygenation index (OI) on day one of life (BOI-d1), where $OI=\frac{F_{i}O_{2}\times MAP}{P_{a}O_{2}}$ has emerged as the most useful metric for the physiological impact of lung hypoplasia and resulting need for respiratory support [75,76,77,78]. In single- and multi-centre studies, BOI-d1 outperformed other perinatal measures (e.g., birth weight, gestational age, LHR) and scoring systems as a predictor of survival and ECMO requirement [79]. A single-centre study (survivors (*n* = 44) and non-survivors (*n* = 24)) found that the trend of serial OIs in the first 48 h of life was a marker of response to respiratory support. Neonates with rising or persistently high OIs were significantly more likely to be deemed unfit for surgery after joint assessment by the surgical and neonatology teams [80]. In one study, a preoperative OI below three was associated with reduced duration of MV and shorter NICU stay and could be used to optimise the timing of surgical intervention [81].

### 3.3. Preoperative Respiratory Support

Retrospective single-centre studies have shown reduced mortality and ECMO use with shorter NICU stays in CDH neonates supported by gentle ventilation with spontaneous initiation of breaths and permissive hypercapnia as the standard of care [82,83,84,85]. The current CDH EURO recommendation is to maintain patients on conventional MV with initial settings of PIP below 25 cmH_2_O and PEEP of 3–5 cmH_2_O at 40–60 breaths/min and a target p_a_CO_2_ range between 50 and 70 mmHg [86]. This follows from the VICI RCT, which showed similar mortality and BPD rates between CDH infants started on conventional MV or HFOV, but found conventional MV to be superior for the secondary outcomes of ECMO requirement, duration of MV, and need for pulmonary vasodilators [87]. In a randomised cross-over trial (*n* = 9), a tidal volume target of 5 or 6 mL/kg was associated with a lower work of breathing in CDH neonates compared to lower target volumes [88]. HFOV remains a rescue option for infants failing to maintain target S_a_O_2_ when ventilated with PIP > 30 cm H_2_O at 60 breaths/min, while failure of oxygenation on HFOV with MAP > 16 cm H_2_O and delta pressure of 30–40 cmH_2_O prompts consideration of ECMO [89]. Use of surfactant has not been shown to improve survival or rates of chronic lung disease and is thus not recommended [90,91,92]. Adjuncts to MV, such as Heliox and liquid ventilation, have only been investigated in small single-centre studies [93,94].

Pulmonary vasodilators such as inhaled nitric oxide (iNO) are commonly used to manage PH in CDH infants [52,95]. A retrospective review of over 3000 CDH patients in the CDH-SG registry found that 62.3% received iNO, including 36.4% of infants without echocardiographic evidence of PH [96]. Inhaled NO may ameliorate PH in cases with elevated RV pressures and preserved LV function but worsen oxygenation in cases with LV diastolic dysfunction [94]. Echocardiographic assessment prior to starting iNO therapy should therefore be mandatory [95,96,97]. Nonetheless, iNO may still fail to ameliorate PH in the absence of LV dysfunction, perhaps due to aberrant pulmonary vascular development, blunting of the NO signalling pathway, and overexpression of vasoconstrictive mediators such as endothelin-1 (ET-1) or the ETA receptor [93]. Evidence on the therapeutic effect of iNO is therefore inconclusive. Both the multi-centre NINOS trial (*n* = 53) and a large retrospective review (*n* = 1777) demonstrated worse survival and need for ECMO after treatment with iNO [98,99]. Single-centre studies, however, reported improved oxygenation with iNO in cases of CDH with atrial or septal right-to-left shunting [100,101]. The use of iNO for management of PH in CDH is included in guidelines by the CDH EURO Consortium but not in those by the American Pediatric Surgical Association (APSA) [86,89]. The CoDiNOS international trial sought to investigate whether intravenous sildenafil or iNO was better at improving outcomes in CDH infants with PH. Unfortunately, the trial failed to recruit enough participants to reach the calculated required sample size [102]. Inotropic agents such as milrinone may also improve oxygenation in CDH infants with PH by improving LV systolic and diastolic function and reducing afterload. A randomised pilot trial of milrinone in CDH is currently being undertaken to determine the safety and feasibility of a large prospective multi-centre trial [103].

### 3.4. Perioperative Considerations

CDH is no longer considered an acute surgical emergency after studies by Breaux et al. showed improved survival (20% to 55%) with delayed repair of CDH after stabilisation of gas exchange and haemodynamics [104,105]. The criteria for preoperative stabilisation in the CDH EURO guidelines include systemic arterial pressures appropriate for gestational age, pre-ductal SO_2_ 85–95% on an F_i_O_2_ < 0.5, lactate < 3 mmol/L, and urine output > 1 mL/kg/h [86]. Some centres recommend waiting until PA pressures have fallen to 70–80% of systemic pressures to lower the risk of postoperative decompensation [106,107]. CDH infants on ECMO may benefit from surgical repair in the first 48 h after cannulation, since retrospective studies observed that delayed CDH repair on ECMO was associated with increased rates of bleeding and longer postoperative ECMO requirements [108,109,110,111].

Surgical repair of CDH can be associated with derangements of arterial blood gas values. In both prospective and retrospective studies, neonates undergoing surgery for CDH were found to develop intraoperative hypercapnia and acidosis, which were more severe during thoracoscopic compared to open repair [31,33]. Intraoperative acid-base disturbance during minimally invasive repair of CDH is thought to be of mixed source, with contributions from both systemic absorption of the insufflating CO_2_ used for thoracoscopy or laparoscopy and metabolic derangements due to transiently reduced perfusion of the viscera as they are returned to the abdomen [33]. In a retrospective study (*n* = 23), HFOV was found to improve CO_2_ elimination and prevent acidosis during thoracoscopic repair of CDH to a greater extent than conventional MV [112].

Respiratory support parameters in CDH patients are also useful in planning the surgical approach. In a retrospective study (*n* = 210), a pCO_2_ above 4.53 kPa (34 mmHg) and the use of HFOV predicted defects too large for primary repair and requiring a prosthetic patch [113]. A multi-centre review (*n* = 235) also observed significantly higher BOI-d1 values in patients requiring patches [79]. In turn, the requirement for a prosthetic patch is a challenge to repair of CDH by thoracoscopy or laparoscopy and is associated with longer durations of MV, higher recurrence rates, and increased mortality [114,115]. The ability to predict a prosthetic repair based on respiratory support requirements is hence useful for parental counselling and may aid in the selection of CDH patients suitable for minimally invasive repair [113,116,117]. In units with the required capacity, CDH repair is carried out by thoracoscopy if a minimally invasive repair is chosen and by laparotomy if an open approach is preferred.

### 3.5. Postoperative Respiratory Support

Surgical repair of the diaphragmatic defect poses a further challenge to respiratory support in CDH infants. Given the need for prolonged invasive MV in many CDH patients, studies have investigated whether neurally adjusted ventilatory assist (NAVA), in which ventilator settings are adjusted based on the recorded electrical activity of the diaphragm (EDi), can facilitate weaning off MV and transition to non-invasive respiratory support [118]. While EDi detection is a challenge in neonates with large diaphragmatic defects, single-centre crossover studies reported clinically significant reductions in PIP, MAP, and F_i_O_2_ after the introduction of NAVA in postoperative CDH infants [119,120]. Small-size feasibility studies also showed that CDH infants could be successfully transitioned from conventional ventilation to invasive ventilation with NAVA and then extubated and supported by NIV with NAVA [121,122]. NAVA was also associated with reduced use of opioids and benzodiazepines, which have a known association with length of NICU stay and mortality in CDH neonates [123]. No study has yet investigated which factors affect the success rate of EDi detection in CDH infants or whether there is a correlation between indices of CDH severity (e.g., LHR) and the likelihood of successfully placing those patients on NAVA.

## 4. Congenital Lung Malformations

**Figure 2:**
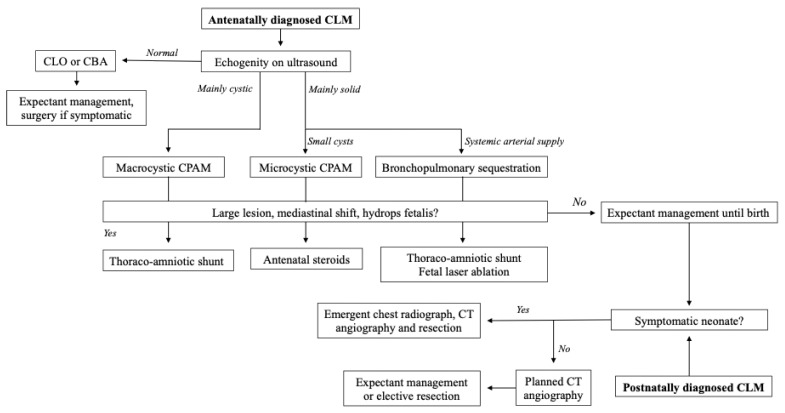
Management strategies in fetuses and neonates with CLMs.

Congenital lung malformations (CLM) are a spectrum of anomalies characterised by aberrant development of the fetal lungs. Most CLMs are congenital pulmonary airway malformations (CPAM), with the rest accounted for by rarer entities such as bronchopulmonary sequestrations (BPS), congenital lobar overinflation (CLO), bronchogenic cysts, and congenital bronchial atresia [124]. Prospective registries of CLMs in countries with universal antenatal screening suggest that the incidence may be as high as 1 in 2500 births [125]. While over 90% of CLM neonates are asymptomatic at birth and may only undergo prophylactic elective resection in later life, those with respiratory distress require urgent surgery in the neonatal period (Figure 2) [126,127].

### 4.1. Antenatal and Preoperative Support

CLMs can be diagnosed on prenatal ultrasonography at 18 to 22 weeks of gestation based on the findings of a cystic or solid space-occupying lesion in the fetal thorax or abnormal size of the fetal lungs [124,128]. The ultrasound-measured CLM volume ratio (CVR), defined as $CVR=\frac{width\times depth\times length\times 0.52}{head\;circumference}$, is a useful predictor of clinical status in symptomatic CLM neonates [129]. These range from mild respiratory distress to hydrops fetalis due to mediastinal shift causing lung hypoplasia and polyhydramnios. In an early prospective study (*n* = 58), a CVR above 1.6 was found to predict hydrops with 75% accuracy [130]. Recent studies have suggested that lower CVR thresholds in the 0.25–1.0 range may aid in the prognostication of outcomes such as respiratory distress and requirement for MV, ECMO, or surgical resection [129,131,132]. In a multi-centre prospective study (*n* = 383), the risk of respiratory distress was shown to be below 10% in both preterm and term-born neonates with CLMs whose prenatal CVR did not exceed 0.4 [133]. A single-centre cohort study (*n* = 80) found that a CVR above 0.39 at 25–30 weeks was predictive of the need for respiratory support in the 24 h after birth and of surgery within two years [134]. A CVR above 0.84 was also found in a retrospective study to predict severe respiratory distress requiring invasive MV (*n* = 89) [135].

CLM patients with evidence of significant mediastinal shift and/or hydrops require planned delivery in a specialist tertiary centre and may also benefit from in-utero treatment to improve neonatal clinical status [136]. Fetuses with large microcystic CPAMs (CVR > 1.6) leading to hydrops may be treated by administration of maternal steroids at 25 to 30 weeks of gestation. These are thought to act by reducing cellular proliferation and fluid production in the lesion, thus enabling compensatory growth of healthy lung parenchyma [137,138]. Single or repeated courses of antenatal maternal steroids have been shown to reduce CVR and improve perinatal mortality in single-centre retrospective studies [139,140,141]. Other studies, however, described more variable responses and suggested that macrocystic CPAMs and BPS may be refractory to antenatal steroids [138]. Moreover, the effect of antenatal steroids on the histology of the lesion and the risk of later pulmonary malignancy is unknown [141]. Fetuses with hydrops secondary to a large macrocystic CPAM may benefit from treatment by serial needle aspiration or ultrasound-guided insertion of a thoraco-amniotic shunt (TAS). Retrospective studies have found that TAS are highly effective at promoting resolution of hydrops and improving survival, with good long-term respiratory outcomes [142,143,144]. Fetuses with BPS leading to massive pleural effusions may be treated by laser coagulation of the systemic feeding vessel. In a multi-centre study (*n* = 12), vascular laser ablation was more effective than TAS insertion in achieving regression of the lesion and reducing sequestrectomy rates in BPS infants [145].

### 4.2. Respiratory Support Strategies in Surgery for CLMs

Postnatal management of CLMs is primarily determined by the clinical status of the patient at birth. CLM neonates with respiratory distress require an urgent chest radiograph to exclude associated pathology (e.g., pneumothorax) and contrast-enhanced CT to characterise the lesion prior to resection [124]. Lobectomy is considered the standard of care, as the boundary between CLMs and healthy lung parenchyma may be difficult to accurately define intraoperatively. Segmental and sub-lobar resections could increase the risk of complications such as persistent pneumothorax [146]. The importance of ensuring complete resection was also emphasised by a retrospective study (*n* = 210), which found that a small subset of CPAMs requiring resection were associated with mucinous adenocarcinoma carrying somatic *KRAS* mutations, which were likely to have already arisen from mucinous cell clusters within CPAMs in utero [147].

The main respiratory challenges during surgery for CLMs are related to the risks of distension of cystic lesions with MV and those of single-lung ventilation. Studies on anaesthesia for CLMs recommend intravenous induction with maintenance of spontaneous breathing and minimal PEEP settings [126]. Inhalational induction with volatile agents (sevoflurane, nitrous oxide) is associated with complications such as failure to intubate and emphysematous distension of CPAM cysts [148,149]. A single-centre RCT (*n* = 110) of children under five years who underwent video-assisted thoracoscopic (VATS) lobectomy or segmentectomy found that lung-protective tidal volume settings (6 mL/kg for two-lung MV, 4 mL/kg for single-lung MV, and PEEP 6 cm H_2_O) improved intraoperative oxygenation and reduced postoperative complications (e.g., pleural effusion, atelectasis, pneumothorax) compared to ‘conventional’ settings (10 mL/kg for two-lung MV, 8 mL/kg for single-lung MV, no PEEP) [150]. Single-lung ventilation in neonates, which requires endobronchial intubation or placement of an extraluminal bronchial blocker, has been shown to be feasible during open or VATS surgery for CLMs [151]. Some authors, however, reported an increased risk of intraoperative hypoxaemia during single-lung ventilation in paediatric patients compared to adults [151]. As a result, some centres elect to avoid single-lung ventilation entirely and maintain visualisation by retracting the ipsilateral lung [152]. In CLM patients with significant respiratory support requirements, lobectomy can be performed while maintaining the patient on HFOV to reduce the risk of air-trapping and distension of the cyst [153]. Early postoperative extubation is recommended to reduce the risk of iatrogenic dehiscence of the bronchial stump due to positive airway pressure [126]. HFOV or ECMO may be required in neonates with CLMs requiring postoperative respiratory support to minimise volutrauma to the surgical site [126,127].

### 4.3. Respiratory Considerations in Asymptomatic CLM Infants

In neonates with prenatally diagnosed CLO or congenital bronchial atresia who only have mild symptoms or are asymptomatic at birth, conservative management is considered appropriate, with surgery indicated only if air trapping or infection develops in the lesion [124]. This approach is supported by a retrospective study (*n* = 20), which showed that most of these patients had evidence of spontaneous clinical and radiological improvement in later life [154].

The appropriate management of asymptomatic CPAMs, BPS, and bronchogenic cysts, however, is controversial. Some paediatric surgeons favour elective resection while others advocate an expectant strategy with surgery only if symptoms emerge [124,155,156]. The rationale for elective resection of asymptomatic CLMs in the first year is to prevent respiratory disease, pulmonary infection, and malignancy in later life [155]. The true incidence of lung tumours in CLM patients, however, remains unquantified and the optimal timing of elective lobectomy in asymptomatic CLM infants is a matter of discussion among paediatric surgeons [157]. Some specialists favour a conservative approach to asymptomatic CLMs to avoid the adverse effects of a potentially unnecessary surgery [156]. The increasing adoption of VATS lobectomy may, however, reduce the musculoskeletal morbidity of elective CLM resections (e.g., chest wall deformity, scoliosis). Furthermore, infants with CLMs who develop symptoms and require expedited surgery have been shown to have worse outcomes compared to those operated on prior to the development of symptoms [158,159]. The design of a cost-effective and safe surveillance program for expectantly managed CLM patients is a challenge due to the risks of repeat exposure to ionising radiation with CT angiography [160]. The CONNECT trial is currently aiming to resolve this controversy by comparing exercise tolerance, pulmonary morbidity, and quality of life at five years between asymptomatic CLM neonates assigned to prophylactic resection versus conservative management [161].

## 5. Anterior Abdominal Wall Defects

Defects of the anterior abdominal wall are a spectrum of anomalies that include gastroschisis and exomphalos (or omphalocele), as well as rarer entities such as prune belly syndrome (PBS), cloacal exstrophy, and pentalogy of Cantrell [162]. This review focuses on gastroschisis and exomphalos, whose prevalences are estimated at, respectively, 1 in 2000 and 1 in 5000 births [163,164]. Gastroschisis and exomphalos are associated with pulmonary complications due to abnormal diaphragmatic function and aberrant lung and chest wall development [165]. In addition, exomphalos is frequently associated with chromosomal abnormalities and congenital syndromes (e.g., trisomy 13, trisomy 18, Beckwith–Wiedemann syndrome) that can contribute to thoracic cage deformity and neonatal respiratory distress [166,167]. The short- and long-term respiratory complications of PBS have been extensively discussed elsewhere and are outside the scope of this review [165,168,169].

### 5.1. Causes of Respiratory Distress in Infants with AWDs

Respiratory distress in infants with AWD reflects underlying pulmonary hypoplasia [165,170]. In a single-centre study of patients with AWD (*n* = 108), Hershenson first reported that neonates with giant (>5 cm) exomphalos were more likely to develop respiratory failure than those with gastroschisis and required longer durations of supplementary oxygen and mechanical ventilation [171]. Comparing chest radiographs, infants with giant exomphalos were found to have smaller chest radiographic thoracic areas (CRTA) than those with small exomphalos or gastroschisis [171]. In addition, the chest walls of patients with giant exomphalos had a distinctive appearance with a narrow chest and downslanting ribs [171]. A later retrospective study (*n* = 12) of the radiographs of stillborn fetuses and neonates showed that exomphalos infants had reduced thoracic volumes [172]. In a single-centre study combining radiographic and post-mortem data (*n* = 114), Argyl found that 42% of infants with giant AWDs had evidence of a thoracic cage deformity with a narrow chest wall and downslanting ribs. Furthermore, measurements including LHR, lung/body weight ratio, and radial alveolar counts showed that infants with giant exomphalos exhibited lung hypoplasia, which was more severe than in infants with small exomphalos or gastroschisis [173]. A single-centre study in infants with exomphalos over 10 cm in diameter (*n* = 20) also found that 70% of patients exhibited lung hypoplasia and 75% required MV and supplemental oxygen for a mean duration of 10 weeks [174].

The size of the abdominal wall defects and the severity of pulmonary hypoplasia are the key prognostic indicators of clinical outcomes in patients with exomphalos. In a single-centre study on exomphalos infants (*n* = 33), liver extrusion into the defect on antenatal ultrasound predicted adverse outcomes including mortality, prolonged NICU stay, and the need for additional interventions [175]. A single-centre study of three-dimensional rotational ultrasound measures in fetuses with exomphalos (*n* = 25) found that fetal lung volume measures correlated significantly with neonatal functional residual capacity (FRC) values as well as the duration of supplemental oxygenation and invasive MV in the NICU [176]. In turn, pulmonary hypoplasia in exomphalos infants has been shown to predict later respiratory morbidity. In a single-centre retrospective study (*n* = 64), the best CRTA on the first day of life was significantly lower in infants with exomphalos than term-born controls. A lower CRTA was significantly associated with the development of BPD at 28 days after adjustment for sex and gestational age [177].

Although gastroschisis is less likely to be associated with preoperative respiratory distress than exomphalos, studies have suggested that gastroschisis patients are also affected by a degree of pulmonary hypoplasia [165]. A single-centre study (*n* = 13) of infants with gastroschisis and exomphalos reported that mean FRC values at a median age of five months were significantly lower than in controls for both groups, indicating impaired antenatal lung growth [178,179]. This was further supported by a study (*n* = 8) which found that mean FRC values on the first day of life and before surgical intervention were significantly below the reference range in both gastroschisis and exomphalos infants [180]. In a single-centre, retrospective study, the median weight-corrected best CRTA on the first day of life in infants with gastroschisis (*n* = 127) was lower than in controls but higher than in infants with exomphalos (*n* = 62). Infants with exomphalos were also significantly more likely to require HFOV, iNO, or postnatal steroids and to develop BPD at 28 days [181].

The reduced prenatal lung growth seen in infants with AWD is thought to result from reduced intrabdominal pressure in utero, leading to poor diaphragmatic function, weak fetal breathing movements, and reduced lung growth [165,173,182]. Additional proposed mechanisms include abnormal shaping of the lower thoracic cage by the configuration of abdominal viscera and aberrant attachment of rectus abdominis to the costal margin leading to downslanting ribs and a narrow chest wall [173,183]. Prenatal analysis of fetuses with AWD demonstrated that lung hypoplasia arises in utero. A study of antenatal ultrasound measures in fetuses with AWD (*n* = 28) showed that the lung/thorax transverse area ratios and chest/trunk length ratios were significantly lower in a subset of patients with giant exomphalos characterised by neonatal death and reduced post-mortem lung/body weight ratio [184]. In addition, a study on fetal MRI-calculated total lung volumes (TLV) in infants with giant exomphalos (*n* = 17) observed that mean age-matched observed/expected (O/E) TLV ratios were lower than in age-matched controls. Fetuses with O/E TLV below 50% were also found to have significantly lower Apgar scores at birth and longer durations of MV, delays to institution of full enteral feeding, and NICU stay [185].

### 5.2. Respiratory Considerations in Surgical Treatment of AWDs

Gastroschisis and exomphalos are amenable to repair by primary or staged techniques as well as non-operative strategies [167]. The choice of approach is dictated by factors including the availability of NICU resources, the size of the defect and amount of herniated viscera, the gestational age of the patient, and the presence of comorbidities [166]. Operative treatment of AWDs involves reduction of the herniated bowel contents into the peritoneal cavity, followed by excision or inversion of the hernial sac (in exomphalos) and sutured closure of the fascia and skin [166,167]. Depending on the degree of visceroabdominal disproportion, however, this approach may result in abdominal compartment syndrome and complications such as intestinal infarction, acute hepatic congestion, respiratory distress, and wound dehiscence [186,187].

The risk of complications due to excessive intra-abdominal pressure after attempted primary closure of gastroschisis or exomphalos in neonates with large AWDs initially led a large proportion of these patients to be treated by staged reduction using a silicone plastic (Silastic) silo [188]. In its original description by Schuster, this approach required general anaesthesia and involved serial reductions of the viscera at the cotside using a silo fashioned by suturing Silastic sheeting to the fascial edges of the defect [188]. The aggressiveness of reductions during staged closure can be gauged based on clinical observations, ventilatory parameters (SO_2_, MAP, EtCO_2_), and/or strategies to monitor intra-abdominal pressure using intragastric or intravesical catheters [166,167,186]. Intra-abdominal pressure should be maintained below 10 mmHg, which represents the recommended threshold for diagnosis of abdominal compartment syndrome in paediatric patients [189].

Advances in neonatal ventilation strategies in the years after the introduction of silos for staged repair of AWDs led to increased survival in infants with high intra-abdominal pressures [166,167]. As a result of this reduction in mortality, urgent primary closure of AWDs in the operating theatre emerged as the preferred treatment strategy in these patients [166,167]. Nonetheless, a large fraction of infants still experienced significant morbidity due to abdominal compartment syndrome and its related complications [166,167]. The importance of controlling intra-abdominal pressures was then revisited with the first description of the sutureless technique for elective delayed reduction of gastroschisis by Bianchi and Dickinson [190]. This approach, in which the viscera are first reduced into the abdomen and then covered by the umbilical cord or a nonadherent dressing to promote granulation over the defect and wound healing, led to more stable cardiorespiratory parameters due to the avoidance of general anaesthesia and muscle relaxants [190]. Later comparative studies demonstrated that sutureless closure of gastroschisis led to similar outcomes to primary fascial repair with reduced respiratory support requirements and intra-abdominal pressures, although some authors reported increased time to institution of enteral feeds and NICU discharge [191,192,193]. Non-operative management is also the preferred option for premature infants with giant exomphalos who cannot tolerate any increases in intra-abdominal pressure due to pulmonary hypoplasia. These patients can be managed conservatively by serial application of ‘escharotic’ dressings such as honey and an overlying corset, aiming to promote epithelialisation of the sac into an umbilical hernia over the course of weeks or months [174,194]. During this time, infants with giant exomphalos who are not requiring respiratory support may be discharged with the dressing applied to the unreduced defect if they can be safely transported home.

Infants in whom operative closure of gastroschisis is preferred over a sutureless technique can also be managed by serial reductions after the application of a preformed spring-loaded silo, which is inserted at the cotside without general anaesthesia. Preformed silos were described in a single-centre comparative study (*n* = 65), which observed that gastroschisis infants treated by delayed sutured repair after placement of a spring-loaded silo had improved fascial closure rates with reduced postoperative complications as well as reduced duration of MV and time to the institution of enteral feeds compared to infants treated by early urgent primary repair [195]. These findings were supported by a single-centre retrospective study (*n* = 53) comparing gastroschisis infants treated by delayed fascial closure after application of a preformed silo and infants treated by traditional approaches including primary fascial closure within six hours of birth or staged reduction under anaesthesia after fashioning of a sutured surgical silo [196]. Infants treated with preformed silos had reduced ventilatory requirements, including lower MAP and F_i_O_2_, higher urine output, and reduced need for inotropes, indicating a reduction in abdominal compartment syndrome [196]. The standardisation of treatment associated with the use of preformed silos is also beneficial to management of gastroschisis in low-resource settings. Alongside standardised protocols for pre-hospital resuscitation (coverage, normothermia, fluids, antibiotics, respiratory, and nutritional support), the use of preformed silos with avoidance of general anaesthesia and primary closure was an essential component of a recently implemented care bundle which led to a marked reduction in mortality from gastroschisis across four African countries (Ghana, Malawi, Tanzania, Zambia) [197,198].

### 5.3. Respiratory Complications Following Treatment of AWDs

Both operative and non-operative treatment of AWDs can be associated with the development of additional respiratory support requirements. This was first reported by single-centre studies in gastroschisis patients, which found that some infants required several days of ventilatory support after reduction of viscera into the abdomen during primary or silo closure [199,200]. The authors postulated that the high intrabdominal pressure caused by the reduction of viscera in infants with major visceroabdominal disproportion led to splinting of the diaphragm and respiratory distress [165,200]. The theory was corroborated by a single-centre study (*n* = 14) measuring respiratory system compliance (C_RS_) in infants with exomphalos and gastroschisis, which found that C_RS_ in both was significantly below reference values before surgery and decreased further in the 48 h after closure of the defect before returning to preoperative values by the third postoperative day [182]. A further study (*n* = 17) in intubated infants with gastroschisis and exomphalos showed that both C_RS_ and forced vital capacity (FVC) were reduced after closure of the defect relative to control values. One month after surgery, FVC values approached those of controls whilst C_RS_ remained reduced [201]. This suggests that repair of AWDs is associated with a transient respiratory dysfunction superimposed on pre-existent lung function abnormalities due to pulmonary hypoplasia [165,182].

The abnormal pulmonary function and respiratory support requirements of neonates with AWD may persist after the repair of the defect [165]. A single-centre retrospective study in infants with giant exomphalos (*n* = 14) ranging from 1 to 58 months of age who underwent pulmonary function testing during follow-up found that mean FVC and forced expiratory volume in the first 0.5 s (FEV_0.5_) were significantly reduced relative to normal ranges, with normal FEV/FVC ratios and respiratory system conductance. In addition, a subset of the patients had an ongoing requirement for supplemental oxygen or invasive ventilation [202]. This suggests that the severe lung hypoplasia seen in infants with giant exomphalos may be associated with a restrictive pattern of lung function abnormalities that persists beyond the first year of life [165]. The long-term respiratory outcomes of infants with AWD are poorly elucidated. Single-centre studies that followed up infants with small or giant exomphalos into school age and adolescence described good health in most, although a minority of participants reported the development of asthma and recurrent chest infections [202,203]. A study in subjects aged 7 to 18 years (*n* = 18) who had surgery as neonates for repair of major exomphalos or gastroschisis showed some moderate exercise limitation. Time to reach maximal heart rate and maximal oxygen consumption were reduced relative to controls [204]. The authors, however, postulated that this finding was more likely to reflect a lack of fitness rather than a primary cardiopulmonary limitation to exercise [204]. Prospective multi-centre follow-up registries of children with AWDs are required to investigate long-term respiratory health in these patients.

## 6. Conclusions

Neonatal surgical conditions are often associated with the development of respiratory distress and the requirement for respiratory support. This represents a challenging clinical scenario, which necessitates coordinated interventions by neonatologists, anaesthetists, and paediatric surgeons.

This review has highlighted key areas of ongoing discussion, including the appropriateness of non-invasive respiratory support (HHFNC, CPAP, NIPPV) in infants with OA-TOF, the effects of FETO and the choice of pulmonary vasodilators in neonates with CDH, the debate between expectant and surgical management of asymptomatic CLMs, and questions around optimum intervention strategies and long-term respiratory health in children with AWDs. Evidence on respiratory support for neonates with surgical disease is limited by the small sample size of many of the existing studies, which is a consequence of the rarity of some these conditions and may lead to a risk of bias. Addressing these limitations will require concerted efforts to design adequately powered prospective trials across neonatal centres internationally and overcome the challenges of patient recruitment, protocol standardisation, and lack of equipoise.
